# Why do many young cancer survivors forgo human papillomavirus vaccination?

**Published:** 2023-01

**DECEMBER 19, 2022 -** Although young cancer survivors face an elevated risk for later developing cervical, oropharyngeal, and several other human papillomavirus (HPV)-related cancers, HPV vaccination rates among this group remain low. This is primarily because of certain vaccine-related concerns, according to a study published by Wiley online in CANCER, a peer-reviewed journal of the American Cancer Society.

To understand why young cancer survivors often opt out of HPV vaccination - which is highly effective in preventing infection with the HPV types most commonly associated with cancer - Brooke Cherven, PhD, MPH, RN, CPON, a researcher at Winship Cancer Institute of Emory University and the Aflac Cancer & Blood Disorders Center of Children’s Healthcare of Atlanta and Emory University, and assistant professor in the Department of Pediatrics at the Emory University School of Medicine, and her colleagues analyzed data from an open-label clinical trial of the HPV vaccine among cancer survivors who were 9-26 years old and 1-5 years from the completion of their cancer treatment. Survivors (or their parents) who declined participation in the trial were asked their reasons for declining.

Among 301 survivors who declined participation in the clinical trial, 215 (71.4%) did so for reasons related to the HPV vaccine. Some of these reasons were similar to those reported in the general population, including concerns about vaccine safety (such as “hearing bad things about the vaccine”), importance (such as viewing the vaccine as “unnecessary”), and timing (such as parents preferring to wait to vaccinate until their child is older). Some survivors also expressed issues specific to cancer survivorship, including preoccupation with health conditions related to their previous cancer treatment, concerns that they had already “been through so much” and wished to avoid further medical interventions, and guidance from a healthcare provider to delay or decline the vaccine “because of all the treatment he’s had.”

Findings from this study may help health care providers - both oncologists and primary care clinicians - address many of the concerns that patients and their families have about HPV vaccination.

“The HPV vaccine is an important tool for cancer prevention, particularly for the vulnerable population of cancer survivors,” said Dr. Cherven. “By incorporating messages that address common concerns, health care providers may feel more prepared and confident when recommending the HPV vaccine to survivors in their practice.”

*Full Citation: “Reasons for refusal of the human papillomavirus vaccine among young cancer survivors.” Brooke Cherven, James L. Klosky, K. Elizabeth Keith, Melissa M. Hudson, Smita Bhatia, and Wendy Landier. CANCER; Published Online: December 19, 2022 (DOI: 10.1002/cncr.34521).*

*URL Upon Publication: http://doi.wiley.com/10.1002/cncr.34521*

*Copyright © 2021 The Cochrane Collaboration. Published by John Wiley & Sons, Ltd., reproduced with permission.*

